# Breast Nurse Intervention to Improve Adherence to Endocrine Therapy Among Breast Cancer Patients in South Ethiopia

**DOI:** 10.1093/oncolo/oyac081

**Published:** 2022-05-07

**Authors:** Sefonias Getachew, Adamu Addissie, Edom Seife, Tariku Wakuma, Susanne Unverzagt, Ahmedin Jemal, Lesley Taylor, Andreas Wienke, Eva J Kantelhardt

**Affiliations:** Department of Preventive Medicine, School of Public Health, Addis Ababa University, Ethiopia; Institute of Medical Epidemiology, Biometrics and Informatics, Martin-Luther-University (Saale), Halle, Germany; Department of Preventive Medicine, School of Public Health, Addis Ababa University, Ethiopia; Institute of Medical Epidemiology, Biometrics and Informatics, Martin-Luther-University (Saale), Halle, Germany; Radiotherapy Center, Tikur Anbessa Hospital, Addis Ababa University, Ethiopia; Department of Surgery, Aira General Hospital, Ethiopia; Institute of General Practice and Family Medicine, Center of Health Sciences, Martin-Luther-University Halle (Saale), Germany; American Cancer Society, Atlanta, Georgia, USA; City of Hope National Medical Center, Duarte, CaliforniaUSA; Institute of Medical Epidemiology, Biometrics and Informatics, Martin-Luther-University (Saale), Halle, Germany; Institute of Medical Epidemiology, Biometrics and Informatics, Martin-Luther-University (Saale), Halle, Germany; Department of Gynaecology, Martin-Luther-University, Halle, Germany

**Keywords:** breast cancer, adherence, tamoxifen, breast nurse, peripheral hospitals, Ethiopia

## Abstract

**Introduction:**

Many women in rural Ethiopia do not receive adjuvant therapy following breast cancer surgery despite the majority being diagnosed with estrogen-receptor-positive breast cancer and tamoxifen being available in the country. We aimed to compare a breast nurse intervention to improve adherence to tamoxifen therapy for breast cancer patients.

**Methods and Materials:**

The 8 hospitals were randomized to intervention and control sites. Between February 2018 and December 2019, patients with breast cancer were recruited after their initial surgery. The primary outcome of the study was adherence to tamoxifen therapy by evaluating 12-month medication-refill data with medication possession ratio (MPR) and using a simplified medication adherence scale (SMAQ) in a subjective assessment.

**Results:**

A total of 162 patients were recruited (87 intervention and 75 control). Trained nurses delivered education and provided literacy material, gave additional empathetic counselling, phone call reminders, and monitoring of medication refill at the intervention hospitals. Adherence according to MPR at 12 months was high in both the intervention (90%) and control sites (79.3%) (*P* = .302). The SMAQ revealed that adherence at intervention sites was 70% compared with 44.8% in the control sites (*P* = .036) at 12 months. Persistence to therapy was found to be 91.2% in the intervention and 77.8% in the control sites during the one-year period (*P* = .010).

**Conclusion:**

Breast nurses can improve cost-effective endocrine therapy adherence at peripheral hospitals in low-resource settings. We recommend such task sharing to overcome the shortage of oncologists and distances to central cancer centers.

## Teaser

In Ethiopia, most patients are estrogen-receptor-positive and could benefit from an endocrine therapy such as tamoxifen; however, endocrine therapy is under-utilized in the country. This article describes a trained breast nurse intervention that was implemented to improve adherence to tamoxifen therapy.

**Implications for Practice**

Patients with breast cancer in rural Ethiopia have few options to access systemic therapy. For endocrine sensitive disease, tamoxifen is highly effective, inexpensive, and has few side effects. Uptake and adherence can be a challenge especially in low-resource settings where it is highly underutilized. The results of this study show that trained breast nurses can increase the subjective level of adherence and the persistence to adjuvant endocrine therapy among patients in rural Ethiopia. Such an approach of task-sharing and de-centralizing cancer care can contribute to reducing abandonment to treatment for improved survival in line with the recent WHO Global Breast Cancer Initiative.

## Introduction

Breast cancer is the most common cancer globally, but there are large disparities in outcome in different settings.^1-3^ In Sub-Saharan Africa (SSA), the 5-year relative survival was shown to be only 59%,^4^ while GLOBOCAN 2021 recently revealed that breast cancer is the second most commonly diagnosed cancer in East Africa and the second highest cause of cancer death in women.^1^ In Ethiopia, it is the leading cancer^5,6^ and recent findings have estimated that it accounts for 20.9% (16 133) of all new cancer cases (reaching up to 30.9% in females) and accounted for 17.5% (9061) of all cancer deaths in 2020.^1^ The 5-year metastatic free survival was 72% for early stage and 33% for late stage cases at the National Cancer Center.^7^ In rural western Ethiopia, the 2-year overall survival rate following surgical treatment only was found to be 53%.^8^

Significant challenges exist to the development of breast cancer control programs in low- and middle-income countries (LMICs).^9^ Treatment for breast cancer in low-resource settings is still limited and many of the patients present to services in at late stages, making this a challenge for health services.^3^ A study from SSA countries revealed that an estimated 28%-37% of breast cancer deaths could be prevented through the earlier diagnosis of symptomatic disease and adequate treatment, with a fairly equal contribution of each.^10^ Recently, the World Health Organisation (WHO) established the Global Breast Health Initiative (GBHI) to improve the outcome especially in LMICs.^11^ One of the three goals aims for the completion of treatment in 90% of patients.

Endocrine (eg, Tamoxifen) therapy is one of the most cost effective and affordable therapeutic options for patients with breast cancer,^12^ requiring few specialized professionals and being best utilized if hormone receptor status is available. The benefit of the therapy is well documented, with an up to 10% absolute increase in 10-year survival probability after 5 years of treatment.^12,13^ It is a standard option in high-income countries but underutilized in many LMICs, including Ethiopia. The therapy has been shown to decrease the annual odds of recurrence and death by a relative 39% and 31%, respectively.^14^ However, adherence to therapy in the adjuvant setting is of particular concern.^15^

Despite substantial implications for survival, the level of adherence to endocrine therapy is not always optimal. Tamoxifen non-adherence ranges from 25% to 59%, with a significant decline during follow-up.^16-19^ In Africa, studies in Nigeria and South Africa reported 25% and 36% non-adherence rates for Tamoxifen, respectively.^20,21^ The early discontinuation of endocrine therapy was also reported in almost one-third of the patients^22^ and those who did not complete treatment ranged from 18% to 73%^23-26^ Patient characteristics, illness and therapy, healthcare, and social and economic factors were the most frequently cited reasons for non-adherence to therapy in most settings^14,21,27,28^).

An intervention which is innovative and tailored to improve the adherence to therapy to address a multifactorial challenge is essential.^23,29,30^ The use of an advanced nurse approach^31^ or a trained nurse-based support^19,32-34^ was suggested to improve adherence to endocrine treatment for women with breast cancer. To our knowledge, such an approach has not yet been tested in randomised clinical trials to date. Similarly, involving nurses to facilitate cancer care in remote areas as a task-shifting model was suggested in SSA,^35,36^ due to scarcity of professionals trained in cancer diagnosis and care in the region.^37^

In Ethiopia, more than two-thirds^8,38^ of patients are estrogen-receptor (ER) positive and could possibly benefit from an endocrine therapy such as tamoxifen. However, the drug is underutilised in the country. A study in west Ethiopia documented the adherence of tamoxifen therapy to be 52% in a 1-year follow-up^39^ and poor patient navigation and lack of awareness about the disease were the major reasons for non-adherence to therapy. In our formative assessment prior to this intervention, we also identified the follow-up of therapy after surgery as a challenge. There is apparently limited professional capacity in cancer care^40^ in the country, although mainly in peripheral areas. The use of a trained nurse approach might have considerable effects in improving adherence to therapy, taking the recommendations from our previous study,^39^ the formative assessment and experience from other settings.^32,34,41^ However, there is a need to test the intervention which is tailored to the context to enhance the adherence to therapy, particularly in disparate populations outside of clinical trials and particularly in LMICs, including Ethiopia.^32^

Thus, we implemented a trained breast nurse intervention to deliver a package of services to patients in peripheral hospitals in the country with the aim of improving adherence to tamoxifen therapy during follow-up and compare adherence with control hospitals.

## Materials and Methods

### Study Design

A cluster randomized study design was conducted in 8 hospitals providing breast cancer care in southern Ethiopia. In this study, the clusters are units of randomization defined as a single hospital. A random allocation of 4 hospitals to the intervention and 4 hospitals to the control (usual care) group was performed using a computer running the randomization procedure. Matching was done to the level of hospitals during the random procedure for assessing the independent effect of breast nurse intervention on the level of adherence to endocrine therapy (tamoxifen) among patients with breast cancer over 1-year period.

### The Study Setting

The interventional hospitals were Aira General Hospital, Attat Our lady of Lourdes Catholic Primary Hospital, Butajira General Hospital and Nigsit Eleni Mohamed Referral Hospitals, while the control hospitals were Saint (St.) Lukas Catholic Hospital, Dubo St. Mary Catholic Hospital, Durame General Hospital and Woliyta Sodo Teaching and Referral Hospitals. These hospitals were selected based on a formative assessment on existing breast cancer surgery and pathology services mainly serving rural populations. There were also options for referral for additional diagnostic services as well as limited therapy options through referral to higher level hospitals.

### Study Participants, Recruitment Period and Follow-Up

All newly diagnosed patients with breast cancer who had initial surgery for breast cancer between February 1, 2018 and December 31, 2019 were recruited in all hospitals as an open cohort design. Patients were followed until a minimum of 12 months or until the end of the study on December 31, 2019. We measured adherence to tamoxifen (main outcome) among patients who attended their 6 and 12-month medication-refill appointments. Discontinuation of therapy was assessed as part of the secondary outcome.

### Patient Inclusion and Exclusion Criteria

Newly diagnosed patients with a pathologically confirmed and surgically treated breast cancer with positive or unknown hormone-receptor status during the study period or during the 12 preceding months were included. The treatment recommendation was given by the local surgeon responsible for the overall care. Patients with breast cancer and known negative hormone receptor status, pregnant women, women with any contraindication for tamoxifen (known thrombosis, stroke) and males were excluded.

### Procedure

All patients received monthly tamoxifen during the first 6 months and then every 3 months during the follow-up. They were interviewed at baseline, and 6 and 12 months after surgery using standardised questionnaires including the medication refill report.

### Intervention Group

All nurses (n=21) expected to participate in the study were trained on how to recruit patients, monitor the side effects of tamoxifen and any contraindications, on appropriate registration and the follow-up of patients, the completion of study documentation, and how to administer questionnaires to patients. Among these, interventional “Breast Nurses”^10^ received additional detailed training and had an attachment at the central Radiotherapy Center at the National Referral Hospital in Addis Ababa. Topics included breast cancer presentation, the pathophysiology of breast cancer, empathetic communication skills, how to give medication-reminder phone calls and how to deliver support, advice and patient education. Hence, breast nurses with this additional training delivered a comprehensive package of services to improve the adherence to tamoxifen therapy for the intervention group. The intervention was developed based on review of the recommendations of different studies conducted on adherence support^19,31,34,39^ and the formative study findings conducted in respective hospitals to look at the overall experience and challenges with follow-up care and support. Intervention included education on breast cancer and provision of literacy material, reminder with phone call, additional empathetic counselling and monitoring of medication refill.

### Routine Care (Control Group)

The non-intervention group received the standard care provided by the respective hospitals. Patients received oral information about the disease and their recommended therapy. We involved 11 nurses from this group to recruit patients, monitor refills and compliance and conduct the study interviews during the baseline and follow-up visits.

### Data Collection

All tools were translated from English to Amharic and then back. Sociodemographic and clinical information, awareness of the disease and willingness to take tamoxifen were collected at baseline. Adherence, persistence, discontinuation, referral and death-related information was collected during the follow-up. The collected data were checked for completeness and consistencies by supervisors and the principal investigator through close follow-up. A pre-tested standard questionnaire and drug refill registration forms were used. The pre-test of the tools was conducted at Tikur Anbesa Specialised Hospital with 10% of the sample size; minor corrections were considered.

### Outcome Measurements

The primary outcome was adherence to tamoxifen at a 12-month duration. The secondary outcomes were persistence to therapy and discontinuation to therapy during the follow-up period. Sociodemographic information was assessed at baseline. Adherence was measured using both medication possession rate (MPR), and a simplified medication adherence questionnaire (SMAQ) scale. For the MPR, from the first day of medication received, we considered the number of tablets which the patient had prospectively received at their immediate previous visit, divided by the days since their immediate previous visit. Patients who refilled their drug ≥80% of the time were considered adherent for that time-period^39,42^ and those who refilled it less than 80% of the time were considered non-adherent from the time point of the immediate previous visit for 12-month duration. In this study, we have also included the 6 months duration adherence report as the discontinuation report to our data is only in the first 6-month interval.

The SMAQ scale has been validated for endocrine treatment of patients with breast cancer with 6 questions,^43^ 4 with ‘yes’/‘no’ answers, and 2 with scales. Patients were asked at month 6 and month 12 about the last 4-12 weeks; those with ≥80% score were considered SMAQ-adherent,^42^ otherwise they were considered SMAQ-non-adherent.

Persistence was described as the duration of time between the initiation of therapy and the last dose before discontinuation. Discontinuation was defined when a patient did not have a refill in a 90-day interval in the first 6 months or for 180 days onwards.

### Sample Size

Sample size was calculated based on the 2-sided continuity corrected Chi-square-test (*α* = 0.05) to compare the adherence of 2 independent groups with equal sample sizes. Adherence to tamoxifen in Ethiopia was reported to be 52% during 1 year.^39^ We aimed to increase this adherence to 85%. To detect this difference in adherence, we used a power of 80% with significance level of 0.05 and a 5% non-inclusion rate, including the design effect of cluster randomization with intracluster correlation coefficient = 0.063 in process variables^44^ and with a cluster number of 8. The sample size for each group was 77, making a total of 154 patients (nQuery Advisor 4.0 and Win Pepi Version 11.65). We added an additional 5%, so a total of 162 patients, to account for early patient deaths.

### Data Analysis

Descriptive statistical methods, a 2-sided Chi-square test or Fisher’s exact test were used for description and the comparison of variables and primary outcomes. The Kaplan-Meier test was used to estimate the probability of persistence and overall survival, while the log-rank test was used to compare groups. We used multivariable logistic regression to adjust for variables which had baseline differences between groups and an effect on the outcome to see the effect of the intervention on adherence. In addition to this, a sensitivity analysis was conducted to see the effect of the intervention when those patients who had prior history of tamoxifen therapy were excluded. Epi info version 7 and SPSS version 21 were used for the analysis.

### Ethical Considerations

Ethical approval was obtained from the Institutional Review Board at the College of Health Science Addis Ababa University (064/17/SPH), National Ethics committee, Ethiopia and the Martin-Luther-University Halle-Wittenberg, Germany (Reg No: 2017-142). Informed written consent was obtained from each participant at baseline. The patient data were kept confidential, so analysis was performed using de-identified data.

## Results

### Sociodemographic Profiles

In total, 162 breast cancer patients eligible to take tamoxifen therapy were included. The mean age of the intervention group was 41.8 (SD 11.1), which was similar to the control group 38.5 years (SD 11.1).

**Figure 1.**
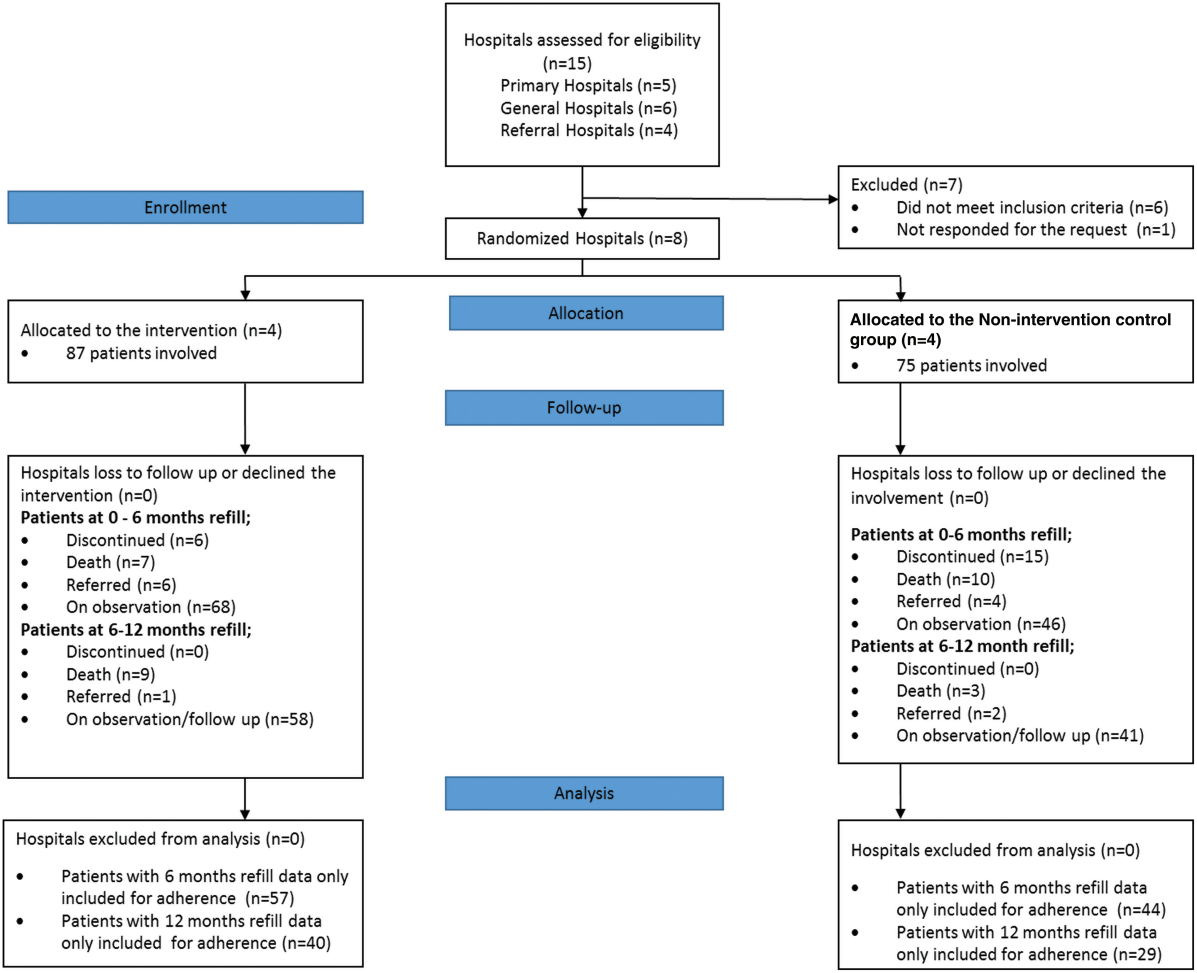
CONSORT flow diagram of progress through the phases of the breast nurse interventional study.

A CONSORT diagram (Fig. 1) shows that of the patients recruited at baseline, 65.5% (57) of the intervention group had a 6-month refill and 46% (40) had a 12-month refill of tamoxifen therapy during the follow-up; a similar pattern was reported in the control group as 58.7% (44) had a 6-month refill and 38.7% (29) had a 12-month refill and were included in the adherence analysis. During the follow-up, 33.3% (29) of patients from the intervention and 45.3% (34) from the control group were right-censored due to death, referral or discontinuation during the first year. Similarly, 20.7% (18) patients from the intervention group and 16% (12) of the control group were left-censored due to their respective refill time period being below 12 months at time of adherence measurement in the 1 year follow-up.

Table 1 describes the sociodemographic profiles of the patients at baseline in the 2 groups. The groups were similar, apart from religion (the proportion of Muslim and Protestant religions varied between groups) and household average annual income, but the latter is a highly subjective response. We found that the majority of patients in both groups (72.4% (63) and 61.3% (46)) were living in a rural setting in the respective areas. Distance to hospitals showed that more than two thirds of patients had ≤30 km to travel from their home to the hospital.

**Table 1.** Sociodemographic profiles of the patients at baseline among groups during breast nurse intervention.

Characteristics	Intervention group	Control group
	(*n* = 87)	(*n* = 75)
	Frequency (%)	Frequency (%)
Age, years		
≤30	18 (20.7)	25 (33.3)
31-40	30 (34.5)	25 (33.3)
41-50	22 (25.3)	18 (24.0)
≥51	17 (19.5)	7 (9.3)
Mean (*+*SD)	41.8 (11.1)	38. 5 (11.1)
Educational status		
Illiterate	55 (63.2)	40 (53.3)
Read and write	8 (9.2)	5 (6.7)
Primary school	10 (11.5)	15 (20.0)
Secondary school	8 (9.2)	11 (14.7)
College level	3 (3.4)	2 (2.7)
University level	3 (3.4)	2 (2.7)
Marital status		
Single	10 (11.5)	5 (6.7)
Married	62 (71.3)	60 (80.0)
Widowed	5 (5.7)	4 (5.3)
Divorced	9 (10.3)	6 (8.0)
Separated	1 (1.1)	0 (0.0)
Occupational status		
Housewife	59 (67.8)	55 (73.3)
Merchant	6 (6.9)	4(5.3)
Government employee	4 (4.6)	7 (9.3)
Self-employed	7 (8.0)	4 (5.3)
Others^a^	11(12.6)	5 (6.7)
Religion		
Orthodox	23 (26.4)	23 (30.7)
Protestant	35 (40.2)	39 (52.0)
Catholic	2 (2.3)	6 (8.0)
Muslim	26 (29.9)	6 (8.0)
No religion	1 (1.1)	1 (1.3)
Husband education		
Illiterate	37 (50.0)	23 (34.3)
Read and write	7 (9.5)	7 (10.4)
Primary school	17 (23.0)	25 (37.3)
Secondary school	4 (5.4)	9 (13.4)
College/university level	9 (12.2)	3 (4.5)
Husband occupation		
Merchant	7 (9.6)	10 (14.9)
Farmer	44 (60.3)	37 (55.2)
Government employee	8 (11.0)	7 (10.4)
Self-employee	5 (6.8)	3 (4.5)
Daily labourer	1 (1.4)	5 (7.5)
Others	8 (11.0)	5 (7.5)
Household average income	Annual (ETB)	
**≤**2500	34 (39.1)	34 (45.3)
2501-5000	9 (10.3)	16 (21.3)
5001-7500	6 (6.9)	0 (0.0)
7501-10 000	3 (3.4)	2 (2.7)
>10 000	35 (40.2)	23 (30.7)
Median (IQR)	3000 (1600, 14 000)	5400 (1500, 18 000)
Place of living		
In town	24 (27.6)	29 (38.7)
Out of town		
	63 (72.4)	46 (61.3)
Distance of hospitals	From home in km	
≤15	26 (29.9)	35 (46.7%)
16-30	33 (37.9)	22 (29.3)
31-45	11 (12.6)	7 (9.3)
46-55	3 (3.4)	3 (4.0)
≥56	14 (16.1)	8 (10.7)
Median (IQR)	20 (10, 36)	17 (10, 30)

Others = student, daily laborer, and private job.

ETB, Ethiopian Birr.

### Treatment and Clinical Characteristics

The clinical profiles of the patients in Table 2 show that most of the clinical variables were similar between the groups; only patients being advised to go to another place for diagnosis or treatment at time of their visit (44.8% of the intervention and 86.7% of the control group), stage at presentation (46.0% were diagnosed late in the intervention group and 70.6% in the control group), FNAC conducted (67.8% in the intervention and 94.7% in the control group), and prior history of using endocrine (tamoxifen) therapy (18.4% in the intervention and 5.3% in the control group) were shown to have a difference.

**Table 2.** Clinical and treatment baseline characteristics of the patients in both groups during breast nurse intervention.

Characteristics	Intervention group	Control group
	Frequency (%)	Frequency (%)
Total visits to hospital till diagnosis		
1-2	40 (46.0)	31 (41.9)
3-5	38 (43.7)	38 (51.4)
≥6	9 (10.3)	5 (6.8)
Mean (SD)	3.02 (1.64)	3.08 (1.82)
Advised to go to other places (for diagnosis or treatment)		
Yes	39 (44.8)	65 (86.7)
No	48 (55.2)	10 (13.3)
Stage at presentation		
I	6 (6.9)	5 (6.7)
II	41 (47.1)	17 (22.7)
III	36 (41.4)	49 (65.3)
IV	4 (4.6)	4 (5.3)
FNAC conducted		
Yes	59 (67.8)	71 (94.7)
No	28 (32.2)	4 (5.3)
Histology result		
Ductal carcinoma	84 (96.6)	68 (90.7)
Lobular carcinoma	3 (3.4)	7 (9.3)
Days interval from diagnosis to surgery in days		
<30	51 (58.6)	41 (54.7)
31-60	11 (12.6)	3 (4.0)
61-90	4 (4.6)	5 (6.7)
≥91	21 (24.1)	26 (34.7)
Median (IQR)	18 (3, 80)	22 (5, 115)
History of using chemotherapy before		
Yes	18 (20.7)	13 (17.3)
No	69 (79.3)	62 (82.7)
History of using radiotherapy		
Yes	6 (6.9)	6 (8.0)
No	81 (93.1)	69 (92.0)
History of using tamoxifen		
Yes	16 (18.4)	4 (5.3)
No		
ER/PR status	71 (81.6)	71 (94.7)
Positive	10 (11.5)	16 (21.3)
Unknown	77 (88.5)	59 (78.7)
HER2 status		
HER2+	4 (4.6)	3 (4.0)
HER2–	6 (6.9)	13 (17.3)
Unknown	77 (88.5)	59 (78.7)
Had comorbidity illness		
Yes	14 (16.1)	8 (10.7)
No	73 (83.9)	67 (89.3)
Type of comorbid illness		
Stroke	3 (21.4)	2 (25.0)
Hypertension	8 (57.1)	3 (37.5)
Heart disease	1 (7.1)	2 (25.5)
Others	2 (14.3)	1 (12.5)

FNAC, fine needle aspiration cytology; ET, endocrine therapy; ER/PR, estrogen progesterone receptor; HER2, human epidermal receptor 2.

### Willingness to Initiate the Therapy and Awareness of the Disease

The willingness to initiate tamoxifen treatment was assessed among patients; overall, 97.75% from the intervention group and 97.3% from the control group expressed their agreement. For awareness of the disease, we found that 89.7% of patients from the intervention group and 94.7% from the control group had information about their disease, and 88.5% from the intervention group and 89.3% from the control group knew that they had breast cancer. Overall, 4.6% of the intervention group and 5.3% of the control group revealed that they had a family history of breast cancer. Our assessment found no major difference between the groups, except for responses to breast cancer being a transmissible disease (6.9% of the intervention and 16.0% of the control group) and breast cancer being detected early (54% of the intervention and 26.7% of the control group; Table 3).

**Table 3.** The willingness to initiate tamoxifen therapy and patient awareness of their disease at the baseline of breast nurse intervention.

Characteristics	Intervention group	Control group
	Frequency (%)	Frequency (%)
Willing to initiate tamoxifen therapy		
Yes	85 (97.7)	73 (97.3)
No	2 (2.3)	2 (2.7)
How much willing to initiate the therapy		
Somewhat willing	3 (3.5)	7 (9.6)
Very much willing	82 (96.5)	66 (90.4)
Do you know about your disease?		
Yes	78 (89.7)	71 (94.7)
No		
How do you name disease you encountered?	9 (10.3)	4 (5.3)
Breast cancer	77 (88.5)	67 (89.3)
Breast infection	5 (5.7)	7 (9.3)
Cervical cancer	3 (3.4)	1 (1.3)
Do not know	2 (2.3)	0 (0.0)
Breast cancer is a transmissible disease?		
Yes	6 (6.9)	12 (16.0)
No	81 (93.1)	63 (84.0)
Perceive breast cancer can be detected early?		
Yes	47 (54.0)	20 (26.7)
No	18 (20.7)	26 (34.7)
I don’t know	51 (31.5)	29 (38.7)
Any one in family had breast problem		
Yes	6 (6.9)	7 (9.3)
No	81 (93.1)	68 (90.7)
Anyone in the family had breast cancer		
Yes	4 (4.6)	4 (5.3)
No	83 (95.4)	71 (94.7)

### Primary Outcome: Adherence to Tamoxifen at One Year

Adherence to tamoxifen therapy was measured as a primary outcome at the 12-month follow-up visit using medication refill data. The measurement was done at 12-month medication refill time for the primary outcome. In our trial, the adherence at 12 months was found to be 90% (36/40) in the intervention group and 79.3% (23/29) in the control group (scored ≥80% on MPR; *P* = .302). Similarly, the level of adherence (scored ≥80% on MPR) at 6 months was 89.5% (51/57) and 79.5% (35/44) in the intervention and control group, respectively (*P* = .164; Table 4).

**Table 4.** Effect of breast nurse intervention on adherence expressed as a proportion of adherent patients using the medication possession ratio and simplified medication adherence questionnaire.

Adherence measures during refill time	Intervention group	Control group	*P*-value
	Frequency (%)	Frequency (%)	
MPR at 12 months			
Adhered	36 (90.0)	23 (79.3)	.302
Not adhered	4 (10.0)	6 (20.7)	
SMAQ at 12 months			
Adhered	28 (70.0)	13 (44.8)	.036
Not adhered	12 (30.0)	16 (55.2)	
MPR at 6 months			
Adhered	51 (89.5)	35 (79.5)	.164
Not adhered	6 (10.5)	9 (20.5)	
SMAQ at 6 months			
Adhered	36 (63.2)	23 (52.3)	.271
Not adhered	21 (36.8)	21 (47.7)	

MPR, medication possession ratio; SMAQ, simplified medication adherence questionnaire.

A SMAQ was used as a secondary outcome to assess the self-reported adherence to observe the subjective behaviour of the patients on adherence level. It has been stated that medication adherence measurement requires more than one approach to describe the patient’s adherence status.^43^ Hence, in our study, where patients were assessed after 12 months of therapy, the level of adherence measured using SMAQ was 70% (28) in the intervention group and 44.8% (13) in the control group (*P* = .036), indicating that the intervention had an effect on the self-reported level of adherence at 12 month duration. Similarly, we had a measurement at 6 months which revealed that 63.2% (36) of the intervention group and 52.3% (23) of the control group showed adherence to tamoxifen therapy (*P* = .271; Table 4).

For further analysis of the self-reported adherence difference at 12 months through the SMAQ, we adjusted for variables which had relevant differences at baseline (Table 5). Variables like stage at presentation, FNAC conducted or not, patients being advised to go another place, history of using endocrine (tamoxifen) therapy and patient’s response to breast cancer being detected early or not were included in the model. The multivariable logistic model revealed that patients in the intervention group were 4 times more likely to have self-reported adherence than in the control group (AOR = 4.05; 95%CI (1.17-14.03)) and the remaining variables showed no strong influence (Table 5). Furthermore, we looked at sensitivity analysis including only patients who did not have a prior history of endocrine therapy (tamoxifen) use and still found a significant difference (*P* = .005) for self-reported level of adherence at 12 months between the groups. The multivariable model analysis also retained a significant effect of the intervention (AOR = 4.90; 95%CI (1.27-18.97)) on the self-reported level of adherence at 12 months, when including patients with no prior history of endocrine therapy use in the model.

**Table 5.** Multivariable logistic model to assess the effect of breast nurse intervention on adherence of endocrine therapy (tamoxifen) during a 12-month refill period.

Variables	Self-reported Adherence status at 12 months		AOR (95% CI)	*P*-value
	Adhered frequency (%)	Not-adhered frequency (%)		
Intervention status				
Intervention group	28 (68.3)	12 (42.9)	4.05(1.17, 14.03)	.028^a^
Control group	13 (31.7)	16 (57.1)	1	
Stage at diagnosis				
I	4 (9.8)	5 (17.9)	1	
II	19 (46.3)	9 (32.1)	1.93 (0.34, 10.94)	.47
III	16 (39.0)	13 (46.4)	0.94 (0.17, 5. 18)	.94
IV	2 (4.9)	1 (3.6)	2.51 (0.14, 45.2)	.53
FNAC conducted				
Yes	32 (78.0)	20 (71.4)	1.55 (0.38, 6.29)	.54
No	9 (22.0)	8 (28.6)	1	
History of using ET				
Yes	5 (12.2)	6 (21.4)	0.37 (0.84, 1.65)	.19
No	36 (87.8)	22 (78.6)	1	
Advised to go to another place				
Yes	26 (63.4)	16 (57.1)	1	.46
No	15 (36.6)	12 (42.9)	0.62 (0.17, 2.25)	
Breast cancer can be detected early				
Yes	20 (48.8)	12 (42.9)	1.73 (0.41, 7.23)	.45
No	13 (31.7)	6 (21.4)	2.59 (0.53, 12.73)	.24
Do not know	8 (19.5)	10 (35.7)	1	

ET, endocrine therapy; AOR, adjusted odds ratio.

### Secondary Outcomes: Discontinuation, Persistence

**Figure 2.**
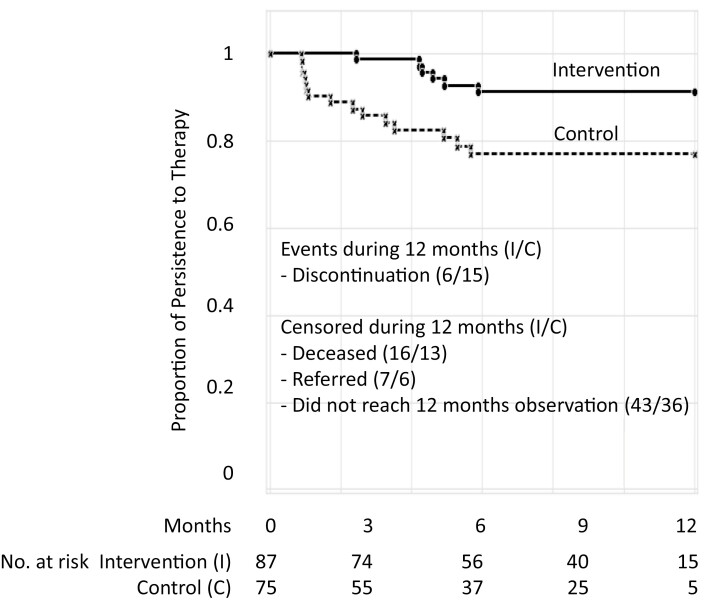
Kaplan-Meier survival curves with time to discontinuation of refilling (months) tamoxifen therapy among groups in a 12-month period.

In our secondary analysis, overall we found that 6.9% (95% CI: 2.9-14.5) of the intervention group and 20% (95%CI: 12.4-30.5) of the control group discontinued tamoxifen therapy during a 1-year period. The level of persistence with the therapy during 12 month was found to be 91.2% in the intervention and 77.8% in the control group. The mean ± SE duration of persistence, as measured by mean time to tamoxifen discontinuation (in months) and by Kaplan-Meier analysis, was 11.3 ± 0.36 months (95% CI, 10.8-11.8) in the intervention group and 9.8 ± 0.5 months (95% CI, 8.9-10.8) in the control group during the 12-month follow-up (*P* = .010; Fig. 2).

## Discussion

This study evaluated a breast nurse intervention which delivered a package of services with the aim of improving level of adherence to tamoxifen therapy among patients with breast cancer. We measured adherence by combining the refill data with a self-reported questionnaire to provide a broad understanding of the adherence behaviour of patients^43^.^45^ The 12 months MPR revealed higher adherence (90%) in the intervention group compared with the control group (79.3%), although no statistical difference was observed (*P* = .302). However, we observed a higher level of medication refill in both groups compared to our previous finding of 52% (39). The results of the SMAQ assessment revealed higher levels of patient adherence to tamoxifen therapy (70%) in the intervention group compared to the control or usual care group (44.8%) during the 1-year period. The adjusted finding revealed that patients from intervention group were 4 times more likely to have self-reported levels of adherence than the control group. However, despite a significant increase in the objectively measured levels of adherence in both groups, there was no difference observed. This might be because the training of data collection nurses on the basics of breast cancer motivated the non-intervention group and helped them to build good communication with patients during their follow-up.

Our findings show that a trained breast nurse intervention had promising effects on improving adherence to adjuvant endocrine (tamoxifen) therapy and was consistent with other studies.^31,33,34^ A previous study reported that nurses are in a good position to assess, monitor and promote adherence to the endocrine therapy, give adequate time to patients and improve communication with them.^31^

A study from Canada also suggested that nurse-led interventions improved patient adherence to oral cancer therapy and follow-up.^46^ One systemic review similarly showed an effect of nurse-led intervention on adherence to prescribed medications.^47^ These findings underscored our hypothesis on the effect of trained nurses on sidestepping levels of non-adherence of the therapy due to multifactorial issues^21,34,39^ and a pragmatic setup. This could contribute to reducing adherence challenges to both the health system and professionals^15^ as part of a task-sharing approach as stated to further support long-term cancer care across the entire health system.^35^

The persistence of therapy after initiation was also statistically significantly higher in the intervention group than in the control group (*P* = .010). The findings also revealed a high level of persistence compared with our previous single-center study (52%) at the 1 year time point.^39^ Importantly, there was a low proportion of discontinuation of therapy among the intervention group compared with the non-intervention group. In other studies, we found higher levels of discontinuation after initiating the therapy,^22,23^ and noted that the early discontinuation of therapy is highly associated with increased recurrence and breast cancer-specific mortality rates, as well higher medical costs.^23,24,48-50^ Similarly, the loss to follow-up is a major issue that threatens the efficacy of treatment protocols, even when treatment is available in most scenarios.^51^ An improved level of persistence with therapy in our intervention group is also a very promising finding for breast nurse interventions with a comprehensive package of services (education on breast cancer, reminder services, empathetic communication and counselling, and further monitoring of drug refills). Other studies have also reported independently improved levels of adherence and persistence^52-54^ in certain regions of the US and France.

The majority of patients in our study were from rural regions and their awareness of the disease was relatively better when initiating therapy since they received information during the diagnostic pathways and surgical care; this is in line with other findings stating that patients who understand their disease are more likely to show compliance with therapy.^55^

In our study, 97.7% of the intervention group and 97.3% of the control group showed willingness to initiate adjuvant tamoxifen therapy. Our study demonstrated that more patients were willing to initiate therapy compared to only 51% in a previous study.^39^ The high willingness and eventual initiation of therapy in our study might be because most patients came from rural areas and a significant number had little education, meaning that they were likely to agree with the suggestions of medical professionals. Another study similarly stated that less educated women were more likely to take tamoxifen, and that their willingness to initiate therapy was not determined by knowledge of risk/benefit or risk perception.^56^ Moreover, patients may have agreed as the drug was available free of charge, and there were few other treatment options, similar to that seen in other African settings.^57,58^

In general, the finding showed that trained breast nurses have shown some promising effects on improving self-reported levels of adherence and persistence with tamoxifen therapy during their 1-year follow-up. The higher level of adherence which was also observed in both groups as per the medication refill report compared with a previous study, although it lacked a significant difference between groups. Hence, to the entire health system this is a very promising opportunity to see a trained nurse support as part of a task-sharing role^35^ for adherence and long-term cancer care support, given that there are limited professionals in cancer diagnosis and care in most African settings, including Ethiopia.^37,40,59^

Our study has certain limitations. First, the low participation of patients during adherence measurement at one year in both groups: 52% of the intervention group and 37.7% of the control group. This may be due to patients exiting the follow-up due to death, referral or discontinuation of therapy during follow-up and also because some were not willing to accept surgery or the adherence support for adjuvant therapy. However, in general we tried to advertise the availability of tamoxifen services in both groups and encouraged women who had a confirmed diagnosis to take the therapy after surgery, followed by recruitment to our respective adherence support group during follow-up.

The second limitation is the inclusion of patients with a prior history of endocrine intake, which might increase an awareness of adherence to therapy. However, we conducted sensitivity analysis by excluding patients with prior experience of taking the therapy before the initiation of the intervention and observed that the effect of the breast nurse intervention was maintained. The third limitation is a lack of greater pathology capacity in the respective settings and patients being referred to other centers during the diagnostic process, as this caused delays to patient recruitment after surgery in some sites during the study period.

Nevertheless, we had strength in implementing a very innovative and feasible intervention tailored to the context. We involved multidisciplinary professionals who had knowledge and skills in breast cancer and adherence support and improved communication to patients. There was also the close supervision in respective hospitals, including the tamoxifen availability in timely manner to the patients during follow up. In addition to these, the formative study was conducted to understand the hospital setups, experiences and challenges in follow-up care in order to shape the intervention which is tailored to the context.

## Conclusion

Our trained breast nurse intervention in Ethiopia improved the self-reported level of adherence and the persistence with tamoxifen therapy among women after breast cancer surgery. Substantial levels of adherence were observed in both groups, as per medication refill data. This indicates that by documenting the patient’s medication refills and having a structured follow-up, including exposure to certain disease- and treatment-related questions, might have brought positive effects on medication adherence to the usual care group.

Our study underpins the positive effect to have a task-sharing role by engaging trained breast nurses improving the follow-up care, including adherence to therapy and timely referral for those in need of further attention. During the study period, we were also able to assure tamoxifen availability in the respective hospitals; from the early inception with formative assessment throughout the process of intervention and supervision. Our findings generally encourage giving more responsibilities to nurses around all other issues related to improve the care of patients. Basic cancer care within the context of emerging non-communicable diseases can become part of the regular nursing curriculum. EHealth solutions can link nurses with higher level professionals to assure supervision and referral if needed. While acknowledging the need for centralized comprehensive cancer centers, we support the idea of a hub-and-spoke model to reach out back and forth to peripheral sites staffed by trained nurses. This will bring basic cancer care within the vicinity of rural patients who are otherwise unable to reach highly specialized centers.^60^ This might have an impact of strengthening the primary healthcare system with strong survivorship care and adherence support to improve patient adherence to treatment as recommended by the BHGI WHO initiative.

## Data Availability

The data underlying this article will be shared on reasonable request to the corresponding author.

## References

[1] Sung H , Ferlay J, Siegel RL, et al. Global cancer statistics 2020: GLOBOCAN estimates of incidence and mortality worldwide for 36 cancers in 185 countries. CA: Cancer J Clin 2021;71:209-249. 33538338 10.3322/caac.21660

[2] Bray F , Ferlay J, Soerjomataram I, et al. Global cancer statistics 2018: GLOBOCAN estimates of incidence and mortality worldwide for 36 cancers in 185 countries. CA: Cancer J Clin 2018;68(6):394-424. 30207593 10.3322/caac.21492

[3] Francies FZ , Hull R, Khanyile R, Dlamini Z. Breast cancer in low-middle income countries: abnormality in splicing and lack of targeted treatment options. Am J Cancer Res 2020;10(5):1568-1591. Available from: https://pubmed.ncbi.nlm.nih.gov/32509398. 32509398 PMC7269781

[4] Joko-Fru WY , Miranda-Filho A, Soerjomataram I, et al. Breast cancer survival in sub-Saharan Africa by age, stage at diagnosis and human development index: A population-based registry study. Int J Cancer. 2020;146(5):1208-1218. 31087650 10.1002/ijc.32406 PMC7079125

[5] Abebe E , Abebe H. Types of cancers diagnosed and the preference of families of adult patients with cancer about disclosing diagnosis to the patients. Ethiop J Health Sci 2017;27(3):255-262. Available from: https://pubmed.ncbi.nlm.nih.gov/29217924. 29217924 10.4314/ejhs.v27i3.7 PMC5614996

[6] Tadele N. Evaluation of quality of life of adult cancer patients attending Tikur Anbessa specialized referral hospital, Addis Ababa Ethiopia. Ethiop J Health Sci 2015;25(1):53-62. Available from: https://pubmed.ncbi.nlm.nih.gov/25733785. 25733785 10.4314/ejhs.v25i1.8 PMC4337080

[7] Kantelhardt EJ , Zerche P, Mathewos A, et al. Breast cancer survival in Ethiopia: a cohort study of 1,070 women. Int J Cancer. 2014;135(3):702-709. 24375396 10.1002/ijc.28691

[8] Eber-Schulz P , Tariku W, Reibold C, et al. Survival of breast cancer patients in rural Ethiopia. Breast Cancer Res Treat. 2018;170(1):111-118. Available from: 10.1007/s10549-018-4724-z. 29479644

[9] Pace LE , Shulman LN. Breast cancer in Sub-Saharan Africa: challenges and opportunities to reduce mortality. Oncologist. 2016;21(6):739-744. 27091419 10.1634/theoncologist.2015-0429 PMC4912363

[10] McCormack V , McKenzie F, Foerster M, et al. Breast cancer survival and survival gap apportionment in sub-Saharan Africa (ABC-DO): a prospective cohort study. Lancet Global Health. 2020;8(9):e1203-e1212. 32827482 10.1016/S2214-109X(20)30261-8 PMC7450275

[11] Anderson BO , Ilbawi AM, Fidarova E, et al. The global breast cancer initiative: a strategic collaboration to strengthen health care for non-communicable diseases. Lancet Oncol. 22:578-581. 33691141 10.1016/S1470-2045(21)00071-1

[12] Anderson BO , Yip C-H, Smith RA, et al. Guideline implementation for breast healthcare in low-income and middle-income countries: Overview of the Breast Health Global Initiative Global Summit 2007. Cancer. 2008;113(S8):2221-2243. 18816619 10.1002/cncr.23844

[13] Plowman PN. Tamoxifen as adjuvant therapy in breast cancer. Current status. Drugs. 1993;46(5):819-833. 7507033 10.2165/00003495-199346050-00003

[14] Early Breast Cancer Trialists’ Collaborative Group. Effects of chemotherapy and hormonal therapy for early breast cancer on recurrence and 15-year survival: an overview of the randomised trials. Lancet 2005;365(9472):1687-1717. 15894097 10.1016/S0140-6736(05)66544-0

[15] Chlebowski RT , Geller ML. Adherence to endocrine therapy for breast cancer. Oncology 2006;71(1-2):1-9. Available from: https://www.karger.com/DOI/10.1159/000100444. 17344666 10.1159/000100444

[16] Rosenberg SM , Stanton AL, Petrie KJ, Partridge AH. Symptoms and symptom attribution among women on endocrine therapy for breast cancer. Oncologist. 2015;20(6):598-604. 25933930 10.1634/theoncologist.2015-0007 PMC4571793

[17] Partridge AH , LaFountain A, Mayer E, et al. Adherence to initial adjuvant anastrozole therapy among women with early-stage breast cancer. J Clin Oncol. 2008;26(4):556-562. 18180462 10.1200/JCO.2007.11.5451

[18] Wheeler SB , Kohler RE, Reeder-Hayes KE, et al. Endocrine therapy initiation among Medicaid-insured breast cancer survivors with hormone receptor-positive tumors. J Cancer Surviv. 2014;8(4):603-610. 24866922 10.1007/s11764-014-0365-3 PMC4970579

[19] Wengström Y. Effectively nursing patients receiving aromatase inhibitor therapy. Breast. 2008;17(3):227-238. 18077168 10.1016/j.breast.2007.11.001

[20] Oguntola AS , Adeoti MI, Akanbi OO. Non-adherence to the use of tamoxifen in the first year by the breast cancer patients in an African population. East Cent Afr J Surg. 2011;16(1).

[21] Du Plessis M , Apffelstaedt JP. Treatment outcomes of breast carcinoma in a resource-limited environment. S Afr J Surg. 2015;53(2):43-47.

[22] Berkowitz MJ , Thompson CK, Zibecchi LT, et al. How patients experience endocrine therapy for breast cancer: an online survey of side effects, adherence, and medical team support. J Cancer Surviv. 2021;15(1):29-39. Available from: 10.1007/s11764-020-00908-5. 32804353 PMC7430212

[23] Wen K-Y , Smith R, Padmanabhan A, Goldstein L. Patient experience of taking adjuvant endocrine therapy for breast cancer: a tough pill to swallow. Patient Exp J. 2017;4(3):104-114.

[24] van Hellemond IEG , Geurts SME, Tjan-Heijnen VCG. Current status of extended adjuvant endocrine therapy in early stage breast cancer. Curr Treat Options Oncol. 2018;19(5):1-18. 10.1007/s11864-018-0541-1 PMC5937869 29704066

[25] Lambert LK , Balneaves LG, Howard AF, Gotay CC. Patient-reported factors associated with adherence to adjuvant endocrine therapy after breast cancer: an integrative review. Breast Cancer Res Treat. 2018;167(3):615-633. 29110151 10.1007/s10549-017-4561-5

[26] Kadakia KC , Kidwell KM, Barton DL, et al. Factors influencing the use of extended adjuvant endocrine therapy. Breast Cancer Res Treat. 2019;175(1):181-189. 30706190 10.1007/s10549-019-05145-8

[27] Murthy V , Bharia G, Sarin R. Tamoxifen non-compliance: does it matter?. Lancet Oncol. 2002;3(11):654. 12424066 10.1016/s1470-2045(02)00895-1

[28] Sedjo RL , Devine S. Predictors of non-adherence to aromatase inhibitors among commercially insured women with breast cancer. Breast Cancer Res Treat. 2011;125(1):191-200. 20495864 10.1007/s10549-010-0952-6

[29] Atreja A , Bellam N, Levy SR. Strategies to enhance patient adherence: making it simple. Medscape Gen Med. 2005;7(1):4. PMC1681370 16369309

[30] American College of Preventive Medicine. Medication Adherence: Improving Health Outcomes Time Tool: A Resource from the American College of Preventive Medicine: American College of Preventive Medicine; 2011 [cited 2021 Mar 24]. Available from: http://www.acpm.org.

[31] Iacorossi L , Piredda M, Marinis MG. Adherence to oral administration of endocrine treat-ment for the patient with breast cancer: a review. Rev Oncol. 2014;2(1):27-32.

[32] Doggrell SA. Adherence to oral endocrine treatments in women with breast cancer: can it be improved?. Breast Cancer Res Treat. 2011;129(2):299-308. 21594663 10.1007/s10549-011-1578-z

[33] Kelly A , Agius CR. Improving adherence to endocrine therapies: the role of advanced practice nurses. Oncology (Williston Park, NY). 2006;20(10 Suppl Nurse Ed):50-4; discussion 55. 18153980

[34] Miaskowski C , Shockney L, Chlebowski RT. Adherence to oral endocrine therapy for breast cancer: a nursing perspective. Clin J Oncol Nurs. 2008;12(2):213-221. 18390458 10.1188/08.CJON.213-221

[35] Stulac S , Binagwaho A, Tapela NM, et al. Capacity building for oncology programmes in sub-Saharan Africa: the Rwanda experience. Lancet Oncol. 2015;16(8):e405-e413. 26248848 10.1016/S1470-2045(15)00161-8

[36] Shulman LN , Mpunga T, Tapela N, et al. Bringing cancer care to the poor: experiences from Rwanda. Nat Rev Cancer. 2014;14(12):815-821. 25355378 10.1038/nrc3848

[37] Adesina A , Chumba D, Nelson AM, et al. Improvement of pathology in sub-Saharan Africa. Lancet Oncol. 2013;14(4):e152-e157. 23561746 10.1016/S1470-2045(12)70598-3

[38] Kantelhardt EJ , Mathewos A, Aynalem A, et al. The prevalence of estrogen receptor-negative breast cancer in Ethiopia. BMC Cancer. 2014;14(1):1-6. 25433805 10.1186/1471-2407-14-895 PMC4258259

[39] Reibold CF , Tariku W, Eber-Schulz P, Getachew S, Addisie A, Unverzagt S et al. Adherence to newly implemented tamoxifen therapy for breast cancer patients in Rural Western Ethiopia. Breast Care 2021. Available from: https://www.karger.com/DOI/10.1159/000512840. 10.1159/000512840 PMC8543345 34720808

[40] Woldeamanuel YW , Girma B, Teklu AM. Cancer in Ethiopia. Lancet Oncol. 2013;14(4):289-290. 23561741 10.1016/S1470-2045(12)70399-6

[41] Ginsburg O , Badwe R, Boyle P, et al. Changing global policy to deliver safe, equitable, and affordable care for women’s cancers. Lancet. 2017;389(10071):871-880. 27814964 10.1016/S0140-6736(16)31393-9

[42] Simon R , Latreille J, Matte C, Desjardins P, Bergeron E. Adherence to adjuvant endocrine therapy in estrogen receptor–positive breast cancer patients with regular follow-up. Can J Surg. 2014;57(1):26-32. 24461223 10.1503/cjs.006211 PMC3908992

[43] Oberguggenberger AS , Sztankay M, Beer B, et al. Adherence evaluation of endocrine treatment in breast cancer: methodological aspects. BMC Cancer. 2012;12(1):1-9. 23066928 10.1186/1471-2407-12-474 PMC3519669

[44] Campbell MK , Fayers PM, Grimshaw JM. Determinants of the intracluster correlation coefficient in cluster randomized trials: the case of implementation research. Clin Trials. 2005;2(2):99-107. 16279131 10.1191/1740774505cn071oa

[45] Finitsis DJ , Vose BA, Mahalak JG, Salner AL. Interventions to promote adherence to endocrine therapy among breast cancer survivors: a meta-analysis. Psycho-Oncol. 2019;28(2):255-263. 10.1002/pon.4959 30511789

[46] Campbell C. Nursing intervention to improve adherence and safety with oral cancer therapy. Can Oncol Nurs J. 2014;24(4):302-309.

[47] Verloo H , Chiolero A, Kiszio B, Kampel T, Santschi V. Nurse interventions to improve medication adherence among discharged older adults: a systematic review. Age Ageing. 2017;46(5):747-754. 28510645 10.1093/ageing/afx076

[48] Van Londen GJ , Donovan HS, Beckjord EB, Cardy AL, Bovbjerg DH, Davidson NE et al. Perspectives of postmenopausal breast cancer survivors on adjuvant endocrine therapy-related symptoms. In: Oncology nursing forum. Vol. 41. NIH Public Access. 2014:660-668. 25355021 10.1188/14.ONF.660-668 PMC4500054

[49] Walker HE , Rosenberg SM, Stanton AL, Petrie KJ, Partridge AH. Perceptions, attributions, and emotions toward endocrine therapy in young women with breast cancer. J Adolesc Young Adult Oncol. 2016;5(1):16-23. 26812461 10.1089/jayao.2015.0051 PMC4779285

[50] Harrow A , Dryden R, McCowan C, et al. A hard pill to swallow: a qualitative study of women’s experiences of adjuvant endocrine therapy for breast cancer. BMJ Open. 2014;4(6):e005285. 10.1136/bmjopen-2014-005285 PMC4067895 24928595

[51] Galukande M , Wabinga H, Mirembe F. Breast cancer survival experiences at a tertiary hospital in sub-Saharan Africa: a cohort study. World J Surg Oncol. 2015;13(1):1-8. 26187151 10.1186/s12957-015-0632-4 PMC4506617

[52] Palmieri FM , Barton DL. Challenges of oral medications in patients with advanced breast cancer. Semin Oncol Nurs. 2007;23(4 Suppl 2):S17-S22. 18054678 10.1016/j.soncn.2007.10.004

[53] Bourmaud A , Rousset V, Collard O, et al. Improving adherence to adjuvant endocrine therapy in breast cancer through a therapeutic educational approach: a feasibility study. Oncol Nurs Forum. 2016;11(3):E94-E103. doi:10.1188/16.ONF.E94-E103 27105205

[54] Davidson B , Vogel V, Wickerham I. Oncologist-patient discussion of adjuvant hormonal therapy in breast cancer. J Commun Support Oncol. 2007;5(3):139-143. 17410813

[55] Chlebowski RT , Kim J, Haque R. Adherence to endocrine therapy in breast cancer adjuvant and prevention settings. Cancer Prev Res. 2014;7(4):378-387. 10.1158/1940-6207.CAPR-13-0389 PMC11649036 24441675

[56] Kaplan CP , Kim SE, Wong ST, et al. Willingness to use tamoxifen to prevent breast cancer among diverse women. Breast Cancer Res Treat. 2012;133(1):357-366. Available from: https://pubmed.ncbi.nlm.nih.gov/22315131. 22315131 10.1007/s10549-012-1960-5 PMC4039196

[57] Kemfang Ngowa JD , Yomi J, Kasia JM, et al. Breast cancer profile in a group of patients followed up at the radiation therapy unit of the Yaounde General Hospital, Cameroon. Obstetr Gynecol Int. 2011;2011:1-5. 10.1155/2011/143506 PMC3140033 21785601

[58] Vanderpuye V , Grover S, Hammad N, et al. An update on the management of breast cancer in Africa. Infect Agents Cancer 2017;12(1):1-12. 10.1186/s13027-017-0124-y PMC5307840 28228841

[59] Joko-Frau Y , Griesel M, Mezger NCS, et al. Breast cancer diagnostics therapy and outcome in sub-Saharan Africa: a population-based registry study. J Natl Compr Canc Netw. 2021;29:1-11. 10.6004/jnccn.2021.7011 34965508

[60] Ayele W , Addissie A, Wienke A, et al. Breast awareness, self-reported abnormalities, and breast cancer in rural Ethiopia: a survey of 7,573 women and predictions of the national burden. Oncologist. 2021;26(6):e1009-e1017. 33650727 10.1002/onco.13737 PMC8176994

